# Massive Periocular Squamous Cell Carcinoma Engulfing the Globe: A Rare Case Report

**DOI:** 10.1155/2014/641086

**Published:** 2014-07-24

**Authors:** Rajender Singh Arora, Anirudh Bhattacharya, Dwarkadas Adwani, Sidak Singh Arora

**Affiliations:** ^1^Department of Head & Neck Oncology, Sujan Surgical Cancer Hospital, Amravati 444601, India; ^2^Department of Oral & Maxillofacial Surgery, VYWS Dental College & Hospital, Amravati 444601, India; ^3^Adwani Multispeciality Dental Hospital, Ambapeth, Amravati 444601, India

## Abstract

The eyelid tumors are rare neoplasms in head and neck oncological practice. The maximum incidence is after sixty years and most of the tumors are situated in the lower eyelid and medial canthus. A wide range of clinical presentations contribute to a high risk of misdiagnosis. The factors such as very large lesions, incomplete excision, histopathologic features such as poor differentiation, multicentric origin, pagetoid spread, and delayed diagnosis are associated with poor prognosis. Because of different tissues at eyelid level, a variety of tumor types and subtypes can arise, but most of them are carcinomas. A rare case of eyelid carcinoma spreading and engulfing the whole of globe which was treated by orbital exenteration and postoperative radiotherapy is presented with a disease-free follow-up of 10 months which, considering its size, is extremely rare. The early diagnosis and proper treatment of such rare tumors still remain the mainstay to predict favourable prognosis.

## 1. Introduction

Malignant eyelid tumors are rare, but they are challenging to recognize and treat, even for experienced oncologist and ophthalmologists. Squamous cell carcinoma (SCC) is an invasive epithelial malignancy showing keratinocytic differentiation. It is the second most common malignant neoplasm of the eyelids comprising 5–10% of all eyelid malignancies [1]. The incidence for eyelid squamous cell carcinoma has been reported to be between 0.09 and 2.42 cases per 1,00,000 population [2]. In the lids, squamous cell carcinoma shows a variety of clinical appearances although it usually presents as a painless, hyperkeratotic lesion that gradually enlarges and eventually ulcerates. There is a tendency for lower lid and lid margin involvement [3]. Recommended local treatment of SCC consists of wide local excision with frozen section. Lymph node metastasis is not commonly seen but, as with our case, for extensively large and fungating growths neck dissection followed by irradiation should be considered.

## 2. Case Report

A male patient aged 42 years reported to the department of maxillofacial surgery with the chief complaint of painless huge growth over left eye and loss of vision. History revealed that the patient was apparently alright 2 years back, when he first noticed a small pea nut size ulcerative growth over left lower eyelid near medial canthus. The growth was painless and persistent in growth. It was not associated with any kind of pain or fever. Initially eye site was normal but as the growth increased his vision started to diminish from left eye due to the increase in the size of growth, approximately 1 year before patient completely loses his vision from left eye. Only due to his extremely poor economic conditions he could not seek any sort of medical treatment.

**Figure 1:**
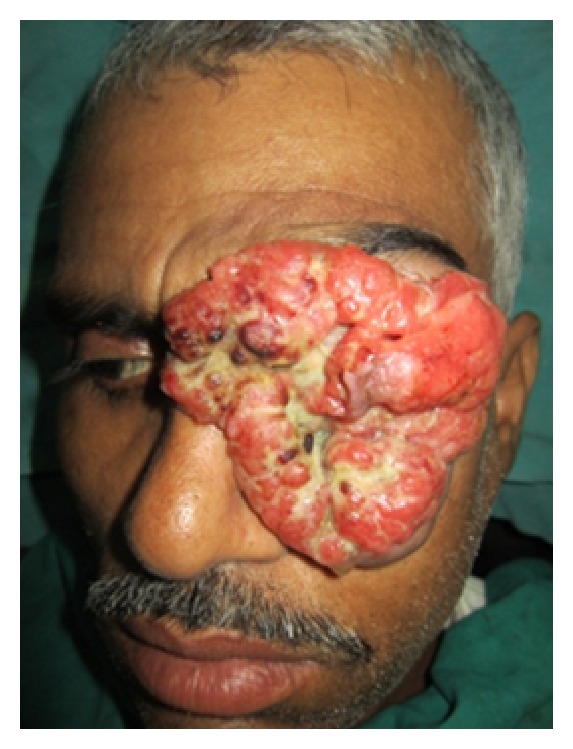
Preoperative lesion.

**Figure 2:**
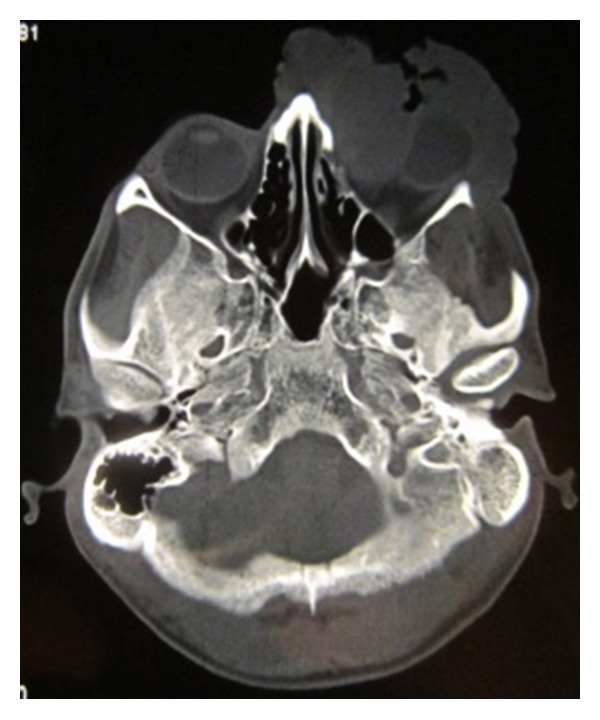
Axial CT scan.

**Figure 3:**
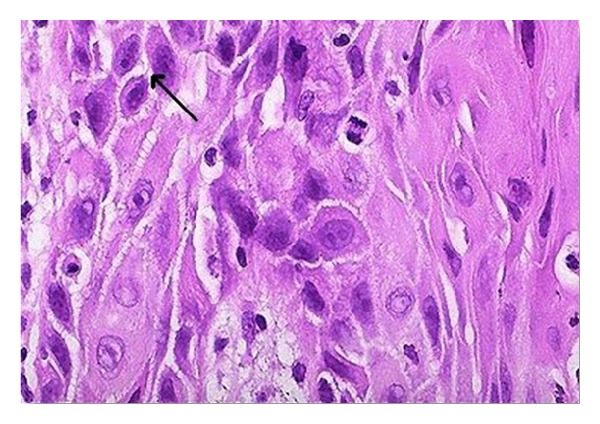
Photomicrograph (H & E stain 20x).

**Figure 4:**
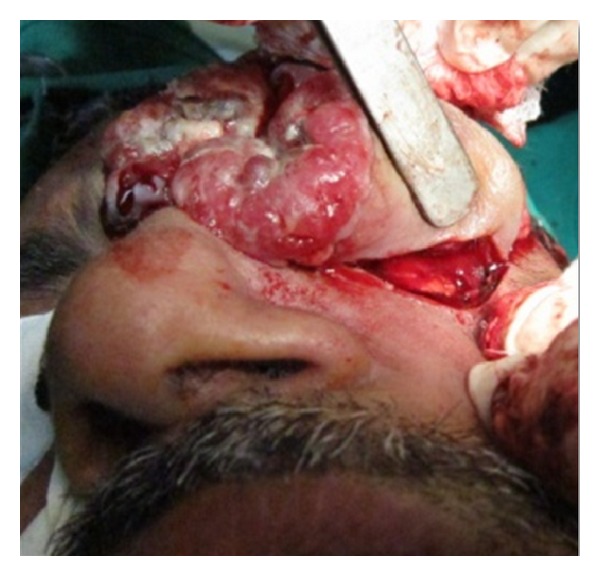
Wide excision of tumor.

**Figure 5:**
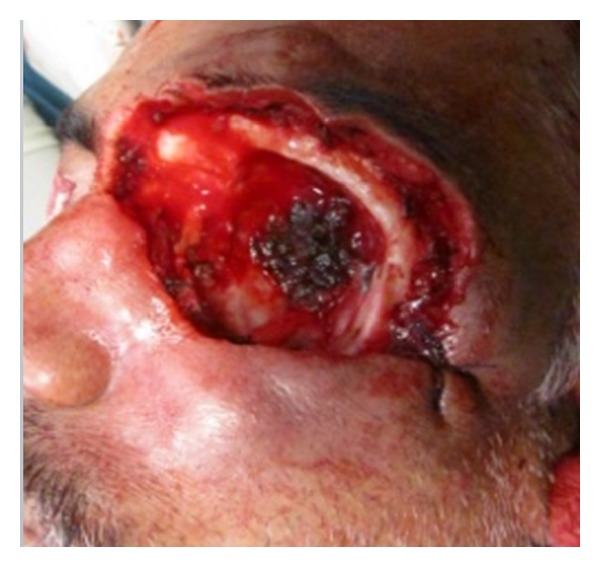
Postexcision.

**Figure 6:**
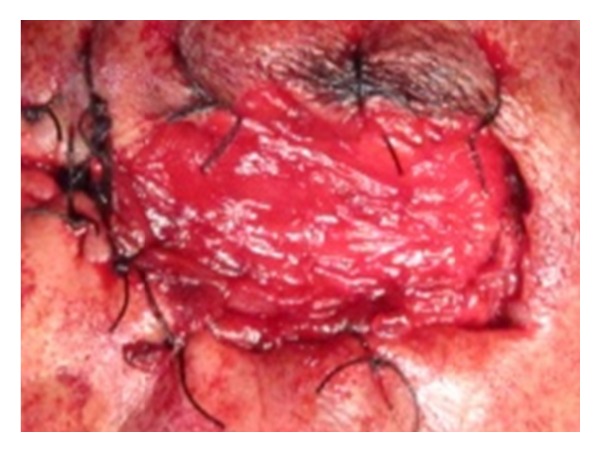
Temporalis muscle flap transpositioned.

**Figure 7:**
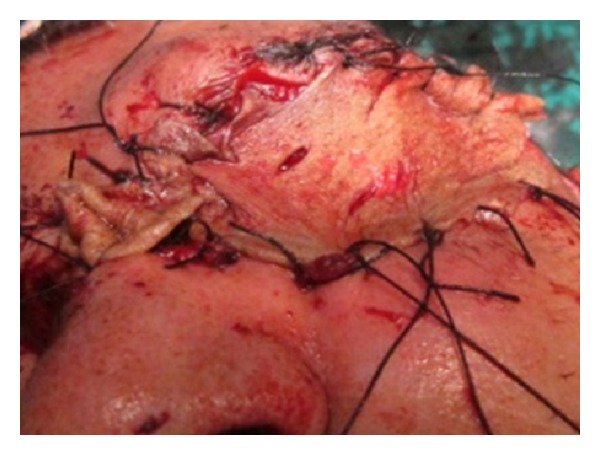
Skin graft.

On clinical examination the growth extended superoinferiorly from supraorbital ridge to ipsilateral alae of nose and mesiodistally from right inner canthus of eye to left side of maxillary buttress crossing midline (Figure 1). Personal history was negative for any detrimental habits. On palpation superficial cervical nodes were not palpable. The growth was ulcerative, exophytic, and fungating and exudates were present. It was approximately 6 × 5 cms in size. The vision from right eye was normal and ocular movements in all directions were intact. All other parameters were within normal range. HR-CT scan was advised. The axial and coronal sections showed large homogenous mass covering the whole left orbit involving the globe but no erosive damage was seen in any of the bony structures (Figure 2). Correlating the clinical history, examination, and radiological findings a provisional diagnosis of carcinoma of left lower eyelid was suggested. Incisional biopsy was performed and histopathological report revealed dermal invasion by abnormal cells from the epidermis, pleomorphism of the tumour cells with presence of keratinization within the cells which give the cells abundant pink cytoplasm, and intraepithelial keratin in the shape of a whorl which confirmed the diagnosis of squamous cell carcinoma of lower eyelid involving the whole of the eye (Figure 3). With all blood investigations repeated and keeping in mind the due risk of surgery in such a massive lesion, wide local excision of the tumour along with removal of all the contents of the respective orbit was planned along with elective neck dissection (Levels I and II). The patient was intubated using orotracheal intubation; skin incision was marked around the tumour leaving 1 cm of healthy looking skin all around which was quite a daunting task looking at the size of the tumor (Figure 4). As the tumour was engulfing the whole of the globe as well as retrobulbar contents with the due consultation with the ophthalmologist, complete orbital exenteration was performed (Figure 5). Elective neck dissection (I-II) performed simultaneously, and all the nodes and resected specimen were sent for the histopathological analysis. Next the pedicled temporalis muscle flap was harvested and was transpositioned medially onto left side of orbit and sutured with skin over the bridge of the nose (Figure 6). Passive tube drain was placed beneath the temporalis muscle flap to drain any collection into the orbit. Split thickness skin graft was harvested from anteromedial side of left thigh approximately 4 × 3 inches and placed over the temporalis muscle flap (Figure 7). The patient showed good compliance and the flap healed uneventfully.

**Figure 8:**
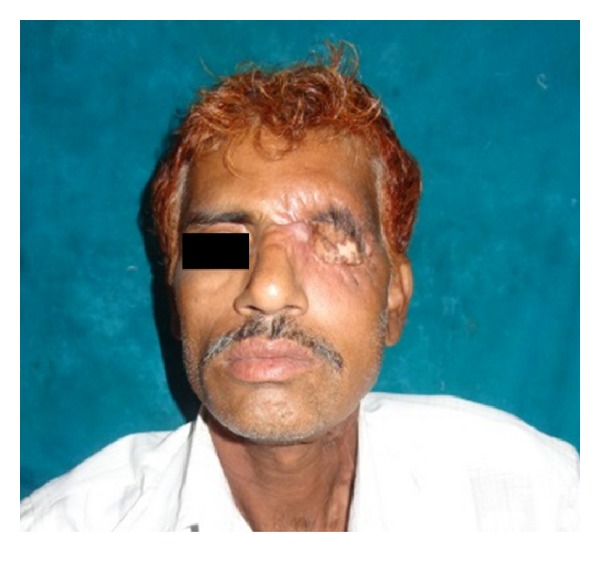
Postoperative healing.

The postsurgical histopathology reports confirmed the tumor-free surgical margins and the preoperative biopsy findings. All the nodes harvested from neck were free from metastasis. The patient was sent for radiotherapy (RT), 70 grays (Gy) for a period of six weeks. The patient is in follow-up for the past 10 months with no signs of recurrence or discomfort (Figure 8). Ocular prosthesis was planned but looking retrospectively at the size of the tumor we still defer ourselves from giving the prosthesis and decided to wait for another 12 months.

## 3. Discussion

Periocular squamous cell carcinoma (SCC) is an aggressive tumor, characterized by perineural involvement and an overall rate of regional lymph node metastases reported to range from 10% to as high as 20% to 25% [4]. Eyelid SCC is a relatively uncommon but potentially fatal disease. It is responsible for considerable morbidity; however, if detected early and treated adequately, the prognosis is generally excellent and death and disability can be reduced [5]. The clinical presentation varies and histological examination is required for accurate diagnosis. Eyelid skin cancers occur most often on the lower lid but may also occur on the upper lid, medial or lateral canthal area, eyebrow, or adjacent eyelid skin (periocular region). They usually appear as painless elevated nodules with or without some ulceration in the center of the growth. At times, rather than a nodule, one sees an indentation or erosion of the skin or eyelid margin. Because of the potential lethality of squamous cell carcinoma, a wider excision than that usually performed for basal cell carcinoma is recommended, as is close postoperative follow-up. The literature suggests that the risk of persistence or recurrence of tumor was increased for patients who delayed seeking medical care after the lesion was first noticed [6]. Research into new immunomodulators will hopefully lead to an increased understanding of the aggressive nature of periocular squamous cell carcinoma and potential aid in the treatment of the tumor. In our present case the massive size of the lesion as well as its extensive growth into the orbit was a very rare scenario. As the patient had completely and irreversibly lost his vision from left eye, therefore it was our only aim to surgically excise the whole tumor with as much clear margins as possible and try to give the patient a chance to survive disease-free. This case is reported to show the massiveness of an eyelid tumor which we could not find in the recent published English literature.
